# Supplementary material for: A Likelihood Approach for Real-Time Calibration of Stochastic Compartmental Epidemic Models
Source: PLoS Comput Biol. 2017 Jan 17;13(1):e1005257. doi: 10.1371/journal.pcbi.1005257 (PMC5240920; doi:10.1371/journal.pcbi.1005257)
Supplement: S1 File — (TAR.GZ) [file pcbi.1005257.s014.tar.gz › HSPH_Online-SI-Revision/IPoi09/n1-mild/n1-mild_BENCH2_table8.pdf]

1	newly 3 weeks spec	9.	10.1481	{1., 22.}	79.4239	0.42842	newly 3 weeks cumul	15.	39.9815	{10., 83.}	180.123	0.367849	newly 5 weeks spec	11.	6.38889	{0., 13.}	68.5185	0.619155	newly 5 weeks cumul	33.	54.9444	{14., 115.}	94.6689	0.265469	newly 47 weeks cumul	75.	76.5741	{16., 159.}	64.1728	0.298149
2	newly 3 weeks spec	1.	1.87302	{0., 4.}	153.968	0.568483	newly 3 weeks cumul	7.	6.90476	{1., 15.}	69.8413	0.457653	newly 5 weeks spec	1.	1.	{0., 3.}	107.937	0.655064	newly 5 weeks cumul	9.	9.09524	{1., 18.}	67.3721	0.45654	newly 43 weeks cumul	9.	12.3333	{1., 27.}	91.0053	0.448273
3	newly 3 weeks spec	7.	3.66667	{0., 14.}	82.0513	1.05854	newly 3 weeks cumul	17.	16.8205	{1., 50.}	95.4751	0.622017	newly 5 weeks spec	1.	1.46154	{0., 4.}	169.231	0.793333	newly 5 weeks cumul	22.	21.0513	{1., 64.}	96.1538	0.65092	newly 52 weeks cumul	22.	25.3077	{1., 82.}	114.103	0.673459
4	newly 3 weeks spec	14.	4.86486	{0., 14.}	76.4479	0.993347	newly 3 weeks cumul	32.	19.5946	{1., 58.}	74.5777	0.649631	newly 5 weeks spec	6.	3.56757	{0., 10.}	79.2793	0.810607	newly 5 weeks cumul	48.	26.9459	{2., 88.}	74.4932	0.691265	newly 46 weeks cumul	263.	39.1351	{2., 132.}	85.5102	1.25346
5	newly 3 weeks spec	5.	6.69231	{2., 13.}	76.3077	0.301176	newly 3 weeks cumul	17.	27.5385	{7., 51.}	86.9683	0.264379	newly 5 weeks spec	1.	3.66154	{0., 10.}	309.231	0.591727	newly 5 weeks cumul	20.	35.7846	{10., 70.}	101.692	0.277295	newly 48 weeks cumul	23.	45.8	{13., 92.}	118.662	0.296154
6	newly 3 weeks spec	3.	15.4107	{5., 26.}	417.262	0.648353	newly 3 weeks cumul	20.	63.0357	{33., 116.}	217.5	0.456009	newly 5 weeks spec	7.	8.05357	{1., 17.}	70.6633	0.317306	newly 5 weeks cumul	35.	82.5179	{38., 158.}	140.255	0.335001	newly 52 weeks cumul	116.	108.786	{42., 226.}	47.8756	0.234837
7	newly 3 weeks spec	5.	9.10345	{1., 20.}	129.655	0.386904	newly 3 weeks cumul	17.	36.431	{11., 76.}	133.367	0.305331	newly 5 weeks spec	6.	4.44828	{0., 8.}	72.9885	0.585237	newly 5 weeks cumul	25.	46.9483	{13., 92.}	115.931	0.296574	newly 51 weeks cumul	45.	57.8276	{13., 128.}	84.1379	0.316395
8	newly 3 weeks spec	13.	18.5102	{8., 35.}	66.562	0.228132	newly 3 weeks cumul	40.	61.1224	{27., 106.}	67.0918	0.210275	newly 5 weeks spec	15.	15.8571	{3., 34.}	57.1429	0.277502	newly 5 weeks cumul	63.	94.0612	{41., 177.}	64.9822	0.207439	newly 43 weeks cumul	262.	208.469	{58., 426.}	52.4926	0.311147
9	newly 3 weeks spec	0.1	15.3333	{5., 34.}	15 233.3	2.08408	newly 3 weeks cumul	7.	54.2917	{20., 104.}	676.19	0.823241	newly 5 weeks spec	0.1	10.25	{3., 20.}	10 150.	1.90239	newly 5 weeks cumul	7.	76.6458	{31., 148.}	994.94	0.971202	newly 42 weeks cumul	7.	127.208	{47., 240.}	1717.26	1.18359
10	newly 3 weeks spec	0.1	4.90476	{1., 10.}	4823.81	1.36688	newly 3 weeks cumul	1.	18.5397	{5., 36.}	1753.97	1.08149	newly 5 weeks spec	0.1	3.36508	{0., 7.}	3300.	1.16742	newly 5 weeks cumul	1.	25.8571	{7., 46.}	2485.71	1.22202	newly 43 weeks cumul	1.	39.1746	{9., 67.}	3817.46	1.38462
11	newly 3 weeks spec	14.	21.9583	{8., 38.}	75.2976	0.24007	newly 3 weeks cumul	36.	69.7708	{32., 110.}	100.058	0.273969	newly 5 weeks spec	13.	17.9167	{5., 37.}	66.9872	0.244715	newly 5 weeks cumul	55.	107.583	{54., 173.}	101.439	0.27712	newly 42 weeks cumul	283.	239.854	{87., 481.}	46.2824	0.253218
12	newly 3 weeks spec	13.	21.2115	{9., 37.}	79.4379	0.243959	newly 3 weeks cumul	24.	69.3846	{35., 109.}	189.423	0.422852	newly 5 weeks spec	5.	16.9038	{5., 28.}	238.846	0.463923	newly 5 weeks cumul	44.	104.25	{51., 172.}	139.904	0.344652	newly 43 weeks cumul	158.	221.808	{87., 414.}	65.3359	0.216669
13	newly 3 weeks spec	2.	2.98276	{0., 8.}	128.448	0.630303	newly 3 weeks cumul	5.	11.8621	{1., 34.}	181.379	0.506753	newly 5 weeks spec	0.1	1.74138	{0., 5.}	1720.69	0.7869	newly 5 weeks cumul	5.	15.569	{1., 42.}	246.552	0.547672	newly 44 weeks cumul	5.	21.4655	{1., 69.}	358.966	0.592741
14	newly 3 weeks spec	0.1	7.38462	{1., 12.}	7300.	1.49026	newly 3 weeks cumul	3.	27.6538	{6., 44.}	821.795	0.736649	newly 5 weeks spec	0.1	3.65385	{0., 8.}	3615.38	1.01521	newly 5 weeks cumul	3.	36.7115	{8., 61.}	1123.72	0.846698	newly 48 weeks cumul	3.	47.1538	{8., 81.}	1471.79	0.934452
15	newly 3 weeks spec	14.	12.9474	{3., 28.}	58.396	0.349392	newly 3 weeks cumul	34.	51.7719	{18., 103.}	79.2054	0.256998	newly 5 weeks spec	7.	8.26316	{1., 21.}	83.208	0.431018	newly 5 weeks cumul	49.	70.2281	{27., 141.}	76.4053	0.262502	newly 48 weeks cumul	104.	97.7368	{35., 202.}	57.1694	0.295717
16	newly 3 weeks spec	6.	11.449	{2., 26.}	130.272	0.396088	newly 3 weeks cumul	34.	45.3061	{9., 98.}	78.0312	0.302192	newly 5 weeks spec	9.	7.4898	{1., 20.}	69.3878	0.460412	newly 5 weeks cumul	52.	62.4286	{10., 141.}	72.4882	0.311537	newly 46 weeks cumul	164.	94.8163	{17., 222.}	58.8352	0.454169
17	newly 3 weeks spec	12.	11.8947	{1., 30.}	76.6082	0.509467	newly 3 weeks cumul	46.	44.0526	{11., 110.}	68.7643	0.361012	newly 5 weeks spec	14.	9.08772	{0., 25.}	73.9348	0.635333	newly 5 weeks cumul	71.	63.0175	{12., 156.}	69.1129	0.393729	newly 49 weeks cumul	150.	101.614	{15., 301.}	72.4444	0.509176
18	newly 3 weeks spec	9.	16.3966	{5., 34.}	100.575	0.289177	newly 3 weeks cumul	36.	55.8276	{25., 100.}	68.7739	0.212393	newly 5 weeks spec	12.	13.3103	{4., 24.}	57.1839	0.276351	newly 5 weeks cumul	56.	83.5	{32., 149.}	67.58	0.217886	newly 42 weeks cumul	105.	159.655	{47., 298.}	79.8686	0.244803
19	newly 3 weeks spec	9.	7.70313	{2., 15.}	52.2569	0.317848	newly 3 weeks cumul	31.	33.4219	{14., 53.}	49.3448	0.213877	newly 5 weeks spec	2.	3.73438	{1., 7.}	128.906	0.425342	newly 5 weeks cumul	38.	43.4219	{17., 74.}	53.9063	0.223259	newly 49 weeks cumul	38.	53.4219	{18., 99.}	67.0641	0.225929
20	newly 3 weeks spec	2.	14.4038	{4., 32.}	625.962	0.754294	newly 3 weeks cumul	25.	52.0769	{17., 105.}	124.154	0.299382	newly 5 weeks spec	0.1	9.36538	{2., 21.}	9269.23	1.7477	newly 5 weeks cumul	27.	73.0577	{22., 141.}	179.131	0.35617	newly 46 weeks cumul	27.	106.942	{30., 212.}	300.214	0.475602
21	newly 3 weeks spec	2.	7.54386	{0., 16.}	310.526	0.60384	newly 3 weeks cumul	9.	26.9649	{4., 62.}	223.782	0.468282	newly 5 weeks spec	1.	4.49123	{0., 11.}	384.211	0.610816	newly 5 weeks cumul	10.	37.2982	{5., 81.}	291.228	0.481362	newly 46 weeks cumul	10.	56.4912	{9., 128.}	474.035	0.574528
22	newly 3 weeks spec	11.	18.2041	{5., 37.}	90.3525	0.277261	newly 3 weeks cumul	38.	61.5714	{26., 110.}	77.1751	0.239121	newly 5 weeks spec	8.	15.6939	{2., 37.}	133.929	0.411656	newly 5 weeks cumul	55.	95.3061	{34., 176.}	90.9462	0.26639	newly 48 weeks cumul	274.	189.673	{46., 475.}	58.275	0.398603
23	newly 3 weeks spec	4.	6.2807	{1., 14.}	101.754	0.361273	newly 3 weeks cumul	24.	23.1228	{6., 53.}	62.2807	0.321586	newly 5 weeks spec	9.	4.10526	{0., 11.}	67.2515	0.68468	newly 5 weeks cumul	45.	32.1754	{8., 72.}	58.8304	0.380645	newly 43 weeks cumul	68.	50.807	{10., 116.}	61.0939	0.396536
24	newly 3 weeks spec	9.	20.5577	{7., 40.}	137.393	0.312013	newly 3 weeks cumul	31.	69.8846	{29., 132.}	130.769	0.309467	newly 5 weeks spec	8.	15.5769	{6., 34.}	111.058	0.298496	newly 5 weeks cumul	48.	101.846	{42., 190.}	118.99	0.289195	newly 45 weeks cumul	112.	170.942	{58., 322.}	75.704	0.230898
25	newly 3 weeks spec	8.	9.91304	{1., 24.}	85.3261	0.434439	newly 3 weeks cumul	29.	36.2609	{6., 78.}	74.063	0.338497	newly 5 weeks spec	12.	6.8913	{0., 15.}	62.8623	0.673132	newly 5 weeks cumul	48.	51.8696	{8., 118.}	70.8333	0.379626	newly 42 weeks cumul	155.	88.	{12., 177.}	58.906	0.513937
26	newly 3 weeks spec	3.	8.61224	{0., 19.}	221.088	0.562409	newly 3 weeks cumul	7.	34.0408	{8., 70.}	392.711	0.618096	newly 5 weeks spec	0.1	5.57143	{0., 12.}	5500.	1.40703	newly 5 weeks cumul	11.	46.5714	{10., 91.}	332.653	0.543394	newly 45 weeks cumul	11.	66.4082	{10., 131.}	510.39	0.639389
27	newly 3 weeks spec	13.	15.6667	{5., 27.}	57.3718	0.236006	newly 3 weeks cumul	30.	54.1042	{22., 91.}	89.7917	0.252176	newly 5 weeks spec	5.	12.6458	{4., 26.}	166.25	0.382128	newly 5 weeks cumul	42.	80.7917	{33., 141.}	101.786	0.272856	newly 42 weeks cumul	51.	144.938	{54., 269.}	190.074	0.406436
28	newly 3 weeks spec	5.	20.4737	{4., 38.}	320.	0.582238	newly 3 weeks cumul	18.	68.0263	{23., 119.}	284.942	0.537653	newly 5 weeks spec	9.	16.4474	{2., 34.}	123.099	0.40384	newly 5 weeks cumul	31.	104.316	{30., 192.}	243.633	0.47982	newly 48 weeks cumul	190.	222.	{55., 465.}	64.5983	0.291374
29	newly 3 weeks spec	0.1	10.7414	{2., 23.}	10 644.8	1.8437	newly 3 weeks cumul	5.	41.0345	{14., 87.}	720.69	0.793562	newly 5 weeks spec	0.1	7.2069	{1., 17.}	7113.79	1.64131	newly 5 weeks cumul	5.	56.8966	{19., 123.}	1037.93	0.931106	newly 45 weeks cumul	5.	81.6379	{24., 163.}	1532.76	1.08114
30	newly 3 weeks spec	8.	6.34426	{2., 12.}	54.7131	0.343367	newly 3 weeks cumul	27.	26.9672	{7., 58.}	56.4663	0.269669	newly 5 weeks spec	5.	4.01639	{0., 9.}	66.2295	0.499583	newly 5 weeks cumul	40.	35.8852	{10., 79.}	54.7131	0.287761	newly 49 weeks cumul	44.	46.6557	{13., 104.}	60.8048	0.274251
31	newly 3 weeks spec	0.1	7.97143	{1., 17.}	7882.86	1.67815	newly 3 weeks cumul	0.1	30.2143	{10., 52.}	30 114.3	2.36656	newly 5 weeks spec	0.1	3.87143	{0., 10.}	3805.71	1.27292	newly 5 weeks cumul	0.1	39.7571	{12., 72.}	39 657.1	2.48208	newly 47 weeks cumul	0.1	52.7429	{13., 97.}	52 642.9	2.59408
32	newly 3 weeks spec	17.	20.3214	{7., 41.}	57.1429	0.24407	newly 3 weeks cumul	43.	64.6429	{28., 107.}	67.5249	0.224822	newly 5 weeks spec	10.	17.0179	{6., 31.}	90.1786	0.294452	newly 5 weeks cumul	62.	100.357	{38., 163.}	77.0161	0.238544	newly 45 weeks cumul	224.	220.464	{70., 450.}	47.0663	0.230529
33	newly 3 weeks spec	3.	17.6471	{5., 32.}	488.235	0.683166	newly 3 weeks cumul	15.	62.3971	{25., 114.}	316.373	0.554432	newly 5 weeks spec	1.	11.2059	{3., 24.}	1023.53	0.929211	newly 5 weeks cumul	19.	87.3676	{34., 160.}	359.985	0.591739	newly 46 weeks cumul	20.	137.338	{51., 251.}	586.691	0.744195
34	newly 3 weeks spec	9.	6.64865	{0., 15.}	75.976	0.702978	newly 3 weeks cumul	32.	22.5135	{2., 49.}	69.5101	0.540996	newly 5 weeks spec	6.	3.78378	{0., 8.}	79.2793	0.767092	newly 5 weeks cumul	42.	31.1622	{2., 72.}	70.9781	0.538103	newly 45 weeks cumul	93.	49.	{2., 123.}	78.5237	0.73276
35	newly 3 weeks spec	10.	19.0938	{8., 33.}	100.938	0.261069	newly 3 weeks cumul	37.	67.2969	{30., 105.}	91.0051	0.243153	newly 5 weeks spec	4.	12.4844	{3., 24.}	224.609	0.424667	newly 5 weeks cumul	48.	95.9063	{41., 156.}	107.422	0.267327	newly 47 weeks cumul	53.	145.422	{60., 256.}	177.034	0.366158
36	newly 3 weeks spec	0.1	5.5942	{1., 13.}	5511.59	1.49817	newly 3 weeks cumul	0.1	23.942	{5., 57.}	23 842.	2.20162	newly 5 weeks spec	0.1	2.37681	{0., 4.}	2331.88	0.985353	newly 5 weeks cumul	0.1	30.2754	{6., 68.}	30 175.4	2.29632	newly 49 weeks cumul	0.1	36.1014	{7., 77.}	36 001.4	2.36659
37	newly 3 weeks spec	5.	8.7037	{1., 19.}	120.	0.422089	newly 3 weeks cumul	27.	32.4444	{7., 77.}	75.583	0.325336	newly 5 weeks spec	7.	5.7963	{0., 13.}	82.8042	0.584846	newly 5 weeks cumul	39.	45.5741	{10., 105.}	74.7863	0.324623	newly 45 weeks cumul	50.	72.7222	{11., 164.}	100.111	0.360218
38	newly 3 weeks spec	2.	6.17188	{1., 15.}	232.031	0.492505	newly 3 weeks cumul	11.	25.4688	{7., 48.}	145.739	0.323649	newly 5 weeks spec	0.1	3.15625	{0., 8.}	3103.13	1.13724	newly 5 weeks cumul	12.	33.2969	{10., 68.}	186.589	0.363731	newly 49 weeks cumul	12.	42.6406	{12., 88.}	259.505	0.437561
39	newly 3 weeks spec	4.	2.96296	{0., 6.}	72.2222	0.596926	newly 3 weeks cumul	11.	13.5185	{3., 26.}	73.7374	0.340069	newly 5 weeks spec	4.	1.74074	{0., 5.}	73.1481	0.822964	newly 5 weeks cumul	27.	17.6296	{3., 37.}	58.7106	0.462588	newly 47 weeks cumul	33.	21.7963	{3., 46.}	58.7542	0.464415
40	newly 3 weeks spec	0.1	5.01613	{0., 13.}	4941.94	1.35301	newly 3 weeks cumul	1.	22.5806	{5., 48.}	2158.06	1.14276	newly 5 weeks spec	0.1	2.37097	{0., 6.}	2335.48	0.935848	newly 5 weeks cumul	1.	28.6774	{5., 60.}	2767.74	1.23998	newly 50 weeks cumul	1.	34.7742	{8., 80.}	3377.42	1.30958
41	newly 3 weeks spec	3.	5.59649	{0., 12.}	145.029	0.493285	newly 3 weeks cumul	11.	21.4912	{4., 39.}	136.842	0.379549	newly 5 weeks spec	0.1	3.29825	{0., 6.}	3264.91	0.971203	newly 5 weeks cumul	11.	29.5789	{4., 48.}	197.608	0.409201	newly 51 weeks cumul	11.	37.9474	{4., 59.}	272.089	0.450405
42	newly 3 weeks spec	0.1	2.41818	{0., 7.}	2394.55	0.904781	newly 3 weeks cumul	0.1	10.6182	{0., 27.}	10 543.6	1.67971	newly 5 weeks spec	0.1	1.25455	{0., 4.}	1281.82	0.521316	newly 5 weeks cumul	0.1	13.4545	{0., 38.}	13 376.4	1.76668	newly 50 weeks cumul	0.1	15.8182	{0., 43.}	15 740.	1.8069
43	newly 3 weeks spec	0.1	1.93939	{0., 6.}	1918.18	0.783459	newly 3 weeks cumul	5.	9.5	{1., 19.}	150.606	0.467338	newly 5 weeks spec	0.1	0.742424	{0., 2.}	778.788	0.397797	newly 5 weeks cumul	5.	11.5	{1., 23.}	181.515	0.461005	newly 52 weeks cumul	5.	12.5909	{1., 25.}	200.303	0.471494
44	newly 3 weeks spec	3.	5.29787	{1., 13.}	124.823	0.446804	newly 3 weeks cumul	3.	23.0638	{5., 49.}	671.631	0.762253	newly 5 weeks spec	0.1	2.93617	{0., 8.}	2878.72	1.13263	newly 5 weeks cumul	3.	30.5319	{6., 70.}	920.567	0.879325	newly 44 weeks cumul	3.	42.0638	{10., 94.}	1304.96	0.998985
45	newly 3 weeks spec	7.	7.32203	{2., 16.}	62.2276	0.30659	newly 3 weeks cumul	21.	26.7797	{9., 51.}	61.5819	0.217383	newly 5 weeks spec	2.	5.49153	{1., 10.}	193.22	0.434424	newly 5 weeks cumul	25.	37.8983	{13., 75.}	73.4237	0.222549	newly 42 weeks cumul	30.	69.5254	{20., 109.}	144.407	0.285991
46	newly 3 weeks spec	13.	12.1132	{2., 27.}	70.6821	0.441183	newly 3 weeks cumul	34.	43.7547	{9., 97.}	77.414	0.325079	newly 5 weeks spec	14.	7.58491	{1., 19.}	69.2722	0.636532	newly 5 weeks cumul	70.	62.	{15., 133.}	63.0189	0.373494	newly 49 weeks cumul	231.	89.3019	{15., 200.}	70.4566	0.657155
47	newly 3 weeks spec	8.	19.1458	{4., 34.}	153.385	0.39332	newly 3 weeks cumul	29.	63.75	{25., 96.}	127.443	0.331991	newly 5 weeks spec	9.	17.5625	{5., 31.}	113.657	0.335836	newly 5 weeks cumul	47.	100.104	{35., 155.}	123.449	0.333321	newly 47 weeks cumul	220.	236.771	{46., 510.}	52.9261	0.261182
48	newly 3 weeks spec	9.	13.7547	{2., 34.}	99.7904	0.408515	newly 3 weeks cumul	23.	47.9811	{12., 104.}	132.075	0.335668	newly 5 weeks spec	13.	9.54717	{2., 26.}	64.8766	0.464396	newly 5 weeks cumul	45.	69.434	{19., 161.}	90.3564	0.296766	newly 42 weeks cumul	52.	110.472	{25., 249.}	133.636	0.342736
49	newly 3 weeks spec	14.	14.9184	{2., 34.}	73.6152	0.406799	newly 3 weeks cumul	26.	49.4082	{9., 123.}	122.998	0.361763	newly 5 weeks spec	13.	10.3469	{1., 27.}	70.3297	0.521108	newly 5 weeks cumul	51.	72.3469	{11., 161.}	88.9156	0.34484	newly 46 weeks cumul	150.	130.082	{13., 386.}	71.5646	0.447395
50	newly 3 weeks spec	9.	12.8983	{3., 28.}	80.6026	0.298804	newly 3 weeks cumul	30.	47.2712	{14., 95.}	83.4463	0.26012	newly 5 weeks spec	4.	8.13559	{1., 21.}	148.305	0.450901	newly 5 weeks cumul	48.	65.6441	{18., 131.}	73.3404	0.25943	newly 43 weeks cumul	70.	104.915	{31., 208.}	81.8402	0.25102
